# Clinical benefit in Phase-I trials of novel molecularly targeted agents: does dose matter?

**DOI:** 10.1038/sj.bjc.6605030

**Published:** 2009-04-28

**Authors:** S Postel-Vinay, H-T Arkenau, D Olmos, J Ang, J Barriuso, S Ashley, U Banerji, J De-Bono, I Judson, S Kaye

**Affiliations:** 1 Royal Marsden Hospital and The Institute of Cancer Research, Drug Development Unit, Downs Road, SM2 5PT, Sutton, UK

**Keywords:** Phase-I trial, molecularly targeted agents, maximal tolerated dose, non-progression rate, clinical benefit

## Abstract

Phase-I trials traditionally involve dose-escalation to determine the maximal tolerated dose (MTD). With conventional chemotherapy, efficacy is generally deemed to be dose-dependent, but the same may not be applicable to molecularly targeted agents (MTAs). We analysed consecutive patients included in Phase-I trials at the Royal Marsden Hospital from 5 January 2005 to 6 June 2006. We considered only trials of monotherapy MTAs in which the MTD was defined. Three patient cohorts (A, B, and C) were identified according to the dose received as a percentage of the final trial MTD (0–33%, 34–65%, >66%). Potential efficacy was assessed using the non-progression rate (NPR), that is, complete/partial response or stable disease for at least 3 months by RECIST. A total of 135 patients having progressive disease before enrolment were analysed from 15 eligible trials. Median age was 57 years (20–86); male : female ratio was 1.8 : 1. Cohort A, B, and C included 28 (21%), 22 (16%), and 85 (63%) patients; NPR at 3 and 6 months was 21% and 11% (A), 50% and 27% (B), 31% and 14% (C), respectively, *P*=0.9. Median duration of non-progression (17 weeks; 95% CI=13–22) was not correlated with the MTD level, *P*=0.9. Our analysis suggests that the potential for clinical benefit is not confined to patients treated at doses close to the MTD in Phase-I trials of MTAs.

The aim of Phase-I trials is to determine the optimal recommended Phase-II dose (RP2D) of a new compound for further clinical investigation. For cytotoxic drugs, this dose traditionally corresponds to the highest dose associated with an acceptable level of toxicity and is derived from clinical data and preclinical dose-toxicity and dose-activity studies (Korn *et al*, 2001). For conventional cytotoxic agents, *in vitro* data and preclinical models using cell lines showed that higher exposure to a drug generally increases tumour cell kill; this dose–response relationship was extrapolated to humans and oncologists have widely adhered to the principle that ‘more is better’. Indeed, recent observations showed that the majority of responses occurred at 75–125% of the RP2D, and that the RP2D was usually the maximal tolerated dose (MTD) (Von Hoff and Turner, 1991; Itoh *et al*, 1994; Parulekar and Eisenhauer, 2004).

Unlike classical chemotherapy agents, new molecularly targeted agents (MTA) are based on different mechanisms and are often considered to be cytostatic rather than cytotoxic. Clinically, this implies that the radiological evaluation of their efficacy by RECIST criteria should include not only a reduction in tumour size, but also non-progression (NP) or disease stabilisation. Therefore, the NP rate (NPR), defined as tumour response plus stable disease (SD), could be a relevant measure of response as well as a potential indicator for clinical benefit.

These novel MTA are often characterised by clinically relevant organ toxicities, which differ from the antiproliferative toxicities seen with cytotoxic chemotherapy, and MTD is not always reached (Booth *et al*, 2008). Thus, the determination of the RP2D based only on toxicity as surrogate marker for activity in the conventional Phase-I setting may be inappropriate for these agents. Moreover, it is unclear whether the RP2D based on toxicity is close to the optimal active dose, as this is derived from a linear dose–efficacy relationship assumption, which may not be relevant for these novel compounds (Parulekar and Eisenhauer, 2004; Cannistra, 2008). Therefore, some authors have advocated establishing a dose range, combining toxicity data – defining the upper limit – and pharmacodynamic and pharmacokinetic data – defining lower dose levels, which could be tested in a randomised Phase-II trial (Booth *et al*, 2008).

Although it is widely agreed that demonstration of anti-tumor activity is not the primary endpoint of Phase-I trials, both patients and physicians hope for benefits from treatment : a discussion about the chances of benefit – that is, tumour response or disease control – is always included in the conversation with Phase-I candidates (Agrawal and Emanuel, 2003). The ethics and individual merits of Phase-I trials have been extensively debated during the ‘era’ of cytotoxic drugs development, and it has been reported that up to 60% of the patients could be treated at sub-therapeutic dose levels (Agrawal and Emanuel, 2003; Koyfman *et al*, 2007). Efforts to minimise the number of patients treated at sub-therapeutic doses and required to reach the MTD have lead to the development of different trial designs such as the continuous reassessment method (Potter, 2002).

The purpose of this study was to investigate whether, in the era of MTA, the chance of benefit in Phase-I trials depended on the dose received, that is, was there any detriment for patients enroled at early dose levels.

## Patients and methods

### Study design, patients, and trials eligibility criteria

This retrospective study considered all consecutive patients evaluable for response and treated in at least one Phase-I trial in the Drug Development Unit at The Royal Marsden Hospital (RMH), United Kingdom, from 5 January 2005 to 6 June 2006. From our database, we selected only patients who were included in trials meeting the following criteria: (1) Trials studying an MTA – an MTA being defined as any agent with any extra- or intracellular target different from those associated with conventional chemotherapy (DNA, tubulin, or cell division machinery); actual tumour shrinkage as opposed to stasis in an experimental model was not a pre-requisite; (2) Trials in which the MTD was described; (3) Trials evaluating an MTA in monotherapy (combinations with conventional chemotherapy or radiotherapy were excluded); and (4) Trials in which the dose escalation method allowed an easy comparison in terms of MTD percentage. All MTD levels were defined individually for each trial on the basis of the protocol MTD definition. All the patients in our analysis had evidence of disease progression before trial entry. Several clinical parameters were collected at study entry, including tumour type, age, sex, ECOG performance status, full blood count, biochemistry (lactate dehydrogenase, and albumin), number and sites of metastasis, and number of earlier systemic cancer treatments. Using these data, we were able to apply our recently validated RMH prognostic score (Arkenau *et al*, 2009).

We analysed the NPR, a combination of complete response, partial response (PR), and SD, at the first assessment after trial entry, and at 3 and 6 months. We considered the NPR at >3 months as a potential indicator for clinical benefit.

All Phase-I trials included in this analysis were approved by the Research and Development Committee and Ethics Committee of the RMH. This analysis received approval of the RMH audit committee.

### Patients’ evaluation and follow-up

All patients underwent regular follow-up and assessment of the disease was carried out by CT (RECIST criteria (Therasse *et al*, 2000) before trial entry, and every 6 to 8 weeks depending on protocol requirements. For prostate cancer patients, PSAWG criteria (Bubley *et al*, 1999) were allowed for the definition of progressive disease, but not for disease response.

### Statistical analysis

Three cohorts (A: 0–33%, B: 34–66%, and C: >67% of the MTD) were defined according to the percentage of the final MTD received by each patient. The percentage of MTD was not considered as a continuous variable, as the distribution of the different dose levels did not follow a regular pattern between 0 and 100% of the MTD. The Kaplan–Meier method and log-rank trend tests were used to compare the NPR, progression-free survival (PFS), and overall survival (OS) for all the patients between the three cohorts with a threshold for significance of *P*=0.05. PFS and OS for all patients were measured from trial entry until documented progression (by RECIST or PSAWG criteria) and death, respectively. For relevant patients, the duration of NP and survival were measured from the first administration of the study drug. The Kendall-*τ* correlation test was used to compare the distribution of the RMH Prognostic Score between the three cohorts. Statistics were carried out using the SPSS-Program (version 15.0, Chicago, IL, USA). The cut-off date for the present analysis was 7 July 2007.

## Results

### Trials Characteristics

**Figure 1:**
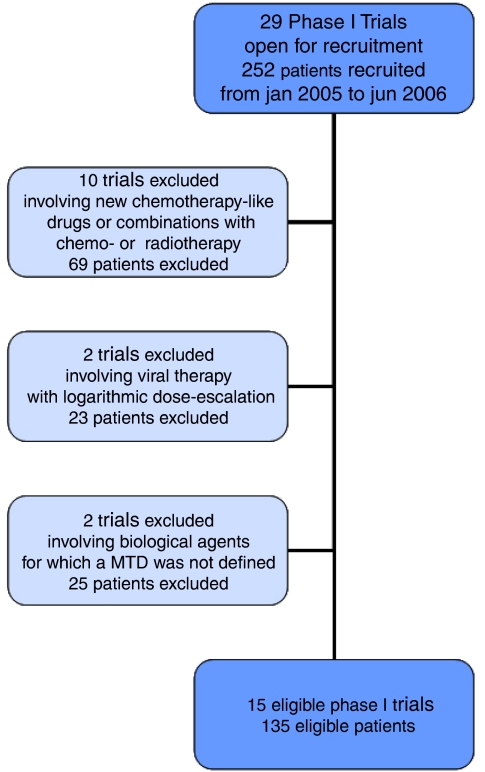
Overview of trial/patient database.

Between 5 January 2005 and 6 June 2006, 29 Phase-I trials (252 patients) were open for recruitment in our unit. We excluded 14 trials (117 patients) based on the earlier defined trial eligibility criteria (Figure 1): 10 trials involved conventional chemotherapy or radiotherapy in combination with an MTA; two trials investigating a virus used a logarithmic dose-escalation scales; two trials involving MTA did not reach the MTD.

The evaluable 15 trials (135 patients) investigated a variety of MTAs including epithelial growth factor receptor inhibitors, anti-angiogenic agents, heat shock protein (HSP90) inhibitors, insulin-like growth factor receptor inhibitors, poly(ADP-ribose) polymerase inhibitors, or epigenetic modulators (Table 1). In total, 12 trials investigated small molecule tyrosine kinase inhibitors. For 12 trials (113 patients), the MTD was defined as ‘the highest dose level below that at which fewer than 2 or more patients experience a DLT’ and for three trials (22 patients), the MTD was defined as ‘the dose at which at least 2 out of 6 patients experience a DLT’. Overall, the median number of dose levels was 5 (range: 4–9).

### Patients’ characteristics

The characteristics of the 135 eligible patients were as follows: the median age was 57 years (range: 20–86) with a male : female ratio of 1.8 : 1. The majority of patients had an ECOG PS of 1 (63%) and only 10% of patients had a PS of 2. The median number of earlier therapies was 2 (range: 0–8) and patients presented with a broad spectrum of tumour types (Table 2).

### Cohorts

Cohorts A, B, and C included 28 (21%), 22 (16%), and 85 (63%) patients, respectively. The larger number of patients included in cohort C is related to the fact that most trials dose expanded at the MTD level. The patients’ characteristics and the RMH prognostic score were not significantly different in the three cohorts reflected by a Kendall-*τ* coefficient of −0.032 (*P*=NS) (Table 3).

### Responses, non-progression rate, and survival

#### Non-progression rate

The median follow-up time of this analysis was 69 weeks. A total of 23 patients (17%) were not evaluable for disease response because of early clinical progression (13 patients), toxicity (five patients including three DLTs), or death (five patients). Nine patients with prostate cancer progressed on PSA measurement according to PSAWG criteria, without radiological progression.

**Figure 2:**
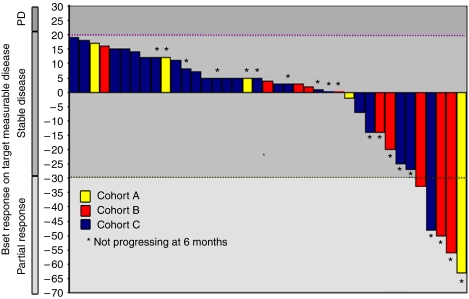
Best response at 3 months (Cohort A, B, and C). This waterfall plot represents the patients remaining progression free during the first 3 months on treatment; the column's colour corresponds to the dose-level cohort.

The NPR for the entire population, at 3 months and 6 months was 32% (43 patients) and 16% (21 patients), respectively. The NPR for the three cohorts at 3 and 6 months was not significantly different, *P*=0.9 (Cohort A: 21% (6 patients) and 11% (3 patients); Cohort B: 50% (11 patients) and 27% (6 patients); Cohort C: 31% (26 patients) and 14% (12 patients), respectively (Table 3). The results of patients with no progression at 3 months are shown as a waterfall plot, in Figure 2. The median duration of NP for all patients was 17 weeks (95% CI: 13–22) (Cohort A: 23 weeks (95% CI: 10–36); Cohort B: 19 weeks (95% CI: 9–30); Cohort C: 15 weeks (95% CI: 12–18)), *P*=0.9. Prolonged NP (>6 months) were seen in patients included in nine different trials, each of them comprising between 4 and 9 dose levels.

#### Responses

Overall, five patients experienced PR (one in cohort A, three in cohort B, and one in cohort C). They were included in three different trials, comprising of 5, 4, and 8 dose levels.

#### Progression-free survival

**Figure 3:**
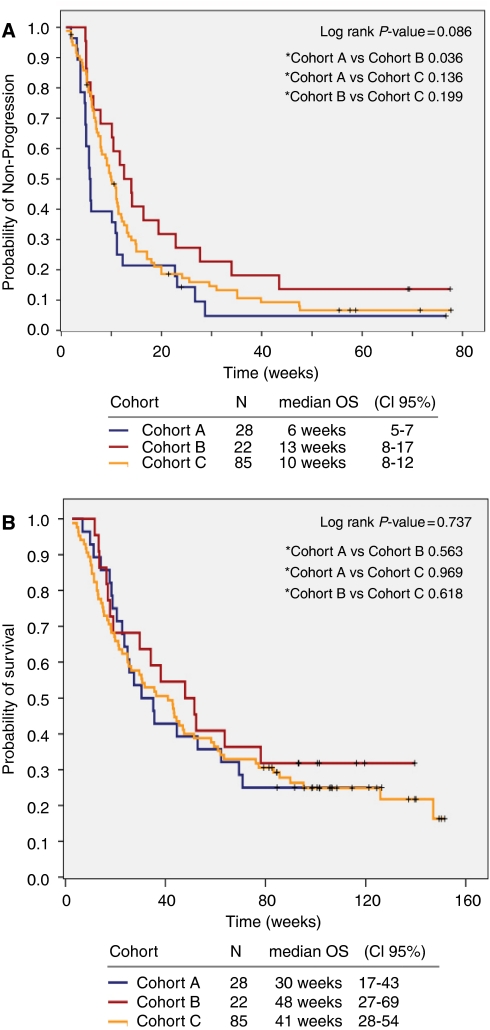
(**A**) Progression-free survival (PFS) of cohort A, B, and C. (**B**) Overall survival (OS) of cohort A, B, and C.

The median PFS was 10 weeks (95% CI: 8–12) for the entire population (Cohort A: 6 weeks (95% CI: 5–7); Cohort B: 13 weeks (95% CI: 8–17); Cohort C: 10 weeks (95% CI: 8–12)), *P*=0.09, Figure 3A.

#### Overall survival

Overall survival was 38 weeks (95% CI: 27–49) for the entire population and was not different between the three cohorts (Cohort A: 30 weeks (CI 95% 18–43); Cohort B: 48 weeks (CI 95%, 28–68); Cohort C: 41 weeks (CI 95% 28–54)), *P*=0.7, Figure 3B.

## Discussion

Phase-I trials have been developed and designed in the era of conventional cytotoxic drug development (Eisenhauer *et al*, 2000). Increasingly, they now include novel MTAs and several challenging questions need to be addressed in this context. Our retrospective analysis investigated whether there was any correlation between the potential for clinical benefit derived from Phase-I treatment and the actual dose that patients received. Patients were divided in three cohorts depending on the percentage of the final MTD of the drug received and we studied the NPR, duration of NP, and response. In summary, we did not observe any statistical differences in the NPR at 3 or 6 months and in the time of NP for the three cohorts. These results support the hypothesis that in the era of Phase-I trials studying MTAs, patients could derive prolonged disease stabilisation, and thus potential for clinical benefit even on lower dose levels; this in turn may influence the way current Phase-I cancer trials are planned, implemented, and analysed.

Our data could also have implications in the way Phase-I trials are discussed with patients before trial entry. Many patients would, if given the choice, prefer being enroled at higher dose levels rather than at lower levels, in which the dose administered is thought to be insufficient. The fact that we could not show any detriment for patients enroled at lower dose levels may result in an enhanced acceptability of entry at initial stages.

In addition, our data emphasise the importance of identifying a biologically active dose in Phase-I trials of MTA (Ratain and Glassman, 2007; Banerji *et al*, 2008; Cannistra, 2008), as this may be significantly different from the MTD (Slaton *et al*, 1999). Clinically, the most effective dose will vary across this range according to the type of agent. Generally, novel MTAs may have a broader therapeutic range compared with conventional cytotoxic agents, and clearly the use of a lower dose could limit the risk of both late and cumulative toxicities using MTAs either as single agents or in combination with conventional chemotherapy or radiotherapy (Cannistra, 2008). At this stage, however, Phase-I trials of MTAs, as well as involving detailed pharmacodynamic and pharmacokinetic analysis, should continue to have the determination of MTD as a primary aim. Subsequent decisions regarding RP2D will vary according to the agent, and randomised Phase-II trials may well be appropriate (Haines, 2008; Sleijfer and Wiemer, 2008).

An alternative conclusion from our study is that the clinical outcome in our patients was not influenced by the Phase-I trial treatment at all. Indeed, this could explain the absence of significant difference in the NPR between the three cohorts, but would not explain the fact that radiological measurable response was seen in some patients, and not exclusively at the MTD level. Nowadays, randomised Phase-II trials evaluating MTAs use, as primary objective, the PFS – or Time To Progression – to determine if the drug deserves further evaluation in a randomised Phase III setting (Korn *et al*, 2001; Ratain and Glassman, 2007). However, we acknowledge that the NPR at 3 and 6 months is a difficult and insufficiently validated measure of clinical benefit and that it would be strengthened by larger numbers, as well as data on symptomatic and/or performance status improvement. A potentially helpful tool to evaluate clinical benefit using PFS was proposed several years ago and entitled the Growth Modulation Index (GMI) (Von Hoff, 1998; Mick *et al*, 2000). The GMI – defined by the ratio TTP(1) : TTP(2), in which TTP(1) and TTP(2) are, respectively, the TTP before and after starting a treatment – compares the rate of change of a tumour lesion before and after Phase-I treatment. It is suggested that a GMI >1.3 reflects treatment effect. Although not yet validated, we applied this methodology to a subgroup of our patients in whom radiological imaging was completely available. In our series, the GMI for patients who experienced disease control for >6 months was 3.1 (range 1.0–9.4), suggesting that these patients had a ‘real’ drug benefit, whereas patients who progressed between 3 and 6 months had a GMI of 1.1 (range 0.3–3.0; 15 patients evaluable). Despite the small patient numbers and the lack of conformity of measurements of tumour progression before Phase-I trial entry, we believe that this methodology deserves further careful evaluation.

A last point to address is the fact that this data set is small and it is possible that the number of patients included in this analysis did not allow us to observe significant differences in dose response. To explore this further, we repeated this analysis by dividing the patients in two groups (> or ⩽50% of the MTD), which allowed us to increase the number of patients per group. Here again, we could not see any statistically significant difference between the two groups with regards to the 3 and 6 months NPR (data not shown). We acknowledge, however, that these data, as they derive from a retrospective single-centre analysis, have to be interpreted cautiously and that our study can only allow the generation of hypotheses. Therefore, we would strongly encourage further validation in larger cohorts.

In summary, our results raise the possibility that the potential for clinical benefit, as measured by NPR and radiological responses, is not confined to patients treated at doses close to the MTD in Phase-I trials of novel MTAs. Whether a prolonged NP leads to a true clinical benefit for the patient is still unclear and deserves further careful evaluation. If validated, this could potentially impact on the discussion between clinicians and patients who are candidates for Phase-I trials. It also reinforces the need to study the biologically effective doses in addition to MTD. Further validation, in larger cohorts and revisiting Phase-I data of MTAs, which have already shown efficacy in Phase-II/III trials, is warranted.

## Figures and Tables

**Table 1:** Trials’ characteristics

**Number of trials**	***N*=15**
**Trials’ characteristics**	***N***
*Drug target*	
EGFR	2
VEGFR2	2
VEGF	1
HSP90	1
IGF-1R	1
HDAC	1
DNA methytransferase	1
PARP	1
TRAIL-R2	1
5*α*-hydroxylase/C17,20lyase	1
Rho-GTP dependant factor	1
Farnesyl transferase	1
Aminopeptidase	1
	
*Class of agent*	
Small molecule TKIs	12
Monoclonal antibodies	2
ASO	1
	
*Number of dose levels*	
4 dose levels	3
5 dose levels	6
6 dose levels	2
8 dose levels or more	4
	
*Dose escalation method*	
Modified fibonacci	1
Accelerated titration	9
Pre-established dose levels increments	5

ASO=antisense oligodeoxynucleotide; EGFR=epithelial growth factor receptor; HDAC=histone deacetylase; HSP90=heat shock protein inhibitor; IGF-IR=insulin-like growth factor receptor inhibitor; PARP=poly(ADP-ribose) polymerase inhibitor; TKIs=tyrosine kinase inhibitors; TRAIL-R2=TNF-related apoptosis inducing ligand-receptor 2; VEGF=vascular endothelial growth factor; VEGFR2=vascular endothelial growth factor receptor 2.

**Table 2:** Patients' characteristics

**Number of patients**	***N*=135**
**Patients’ characteristics**	**Total (%)**
*Age*	
Median (range)	57 (20–86)
	
*Sex*	
Male	87 (64%)
Female	48 (36%)
	
*Performance status (ECOG)*	
0	37 (27%)
1	85 (63%)
2	13 (10%)
	
*Earlier therapy*	
Median (range)	2 (0–8)
	
*Tumour type*	
Breast and gynaecological	24 (18%)
Prostate	21 (16%)
Sarcoma	21 (16%)
Thoracic	20 (15%)
Gastro-intestinal	15 (11%)
Renal	12 (8%)
Melanoma	5 (4%)
Others	17 (13%)
	
*RMH Prognostic score*	
0	16 (12%)
1	38 (28%)
2	51 (38%)
3	30 (22%)

ECOG=Eastern cooperative oncology group; RMH=Royal Marsden Hospital prognostic score.

**Table 3:** Patients’ distribution and MTD cohorts

	**Cohort A**	**Cohort B**	**Cohort C**	**Total**
Number of patients (%)				
	28 (21%)	22 (16%)	85 (63%)	135
Gender (male/female)				
	15/13^*^	13/9^*^	59/26^*^	87/48
Age (median (range))				
	60 (20–83)	56 (27–77)	57 (25–86)	57 (20–86)
*Performance status*				
ECOG 0	6^*^	6^*^	22^*^	34
ECOG 1	17^*^	12^*^	51^*^	80
ECOG 2	5^*^	2^*^	3^*^	10
				
*RMH Prognostic score*				
0–1	10 (36%)^†^	9 (41%)^†^	35 (40%)^†^	54 (40%)
2–3	18 (64%)^†^	13 (59%)^†^	51 (60%)^†^	81 (60%)
				
*Non-progression rate*				
3-month NPR	6 (21%)^‡^	11 (50%)^‡^	26 (31%)^‡^	43/135 (32%)
6-month NPR	3 (11%)^‡^	6 (27%)^‡^	12 (14%)^‡^	21/135 (16%)
				
Objective responses				
	1	3	1	5

ECOG=Eastern cooperative oncology group; MTD=maximal tolerated dose; RMH=Royal Marsden Hospital, NPR=non-progression rate.

^*^P=0.1.

^†^*P*=0.7.

^‡^*P*=0.9.
